# Draft Genome Sequence of Carbapenem-Resistant Pseudomonas fluorescens Strain BWKM6, Isolated from Feces of Mareca penelope

**DOI:** 10.1128/genomeA.00186-18

**Published:** 2018-03-22

**Authors:** Takehiko Kenzaka, Katsuji Tani

**Affiliations:** a Environmental Science and Microbiology, Faculty of Pharmacy, Osaka Ohtani University, Nishikiori-kita, Tondabayashi, Japan

## Abstract

Migratory birds are potential vehicles of antibiotic-resistant bacteria. Here, we isolated the multidrug-resistant Pseudomonas fluorescens strain BWKM6 from the feces of Mareca penelope. The strain’s draft genome sequence indicates that it harbors a metallo-beta-lactamase, a class C beta-lactamase, and several multidrug efflux pumps.

## GENOME ANNOUNCEMENT

Many pathogenic human enteric bacteria have been isolated from wild birds (1). Studies have reported that antibiotic-resistant bacteria travel long distances via migratory birds (2). Owing to their ability to migrate long distances within short time periods, migratory birds are a potential source of antibiotic-resistant bacteria that may be transmitted to humans (3). One migratory bird, the Eurasian wigeon (Mareca penelope), breeds in the northernmost areas of Europe and Asia. The global population of M. penelope is estimated to be approximately 2.8 to 3.3 million (4). M. penelope leaves its breeding grounds during late summer to arrive in autumn at its wintering grounds across Europe and Asia. This species lives primarily in lakes and rivers and along coastlines, and it prefers a location near edible aquatic and terrestrial plants. The number of observed M. penelope individuals in Japan has been hundreds of thousands per year. However, the incidence and types of antibiotic-resistant bacteria associated with migratory birds in East Asia remain unknown.

The carbapenem-resistant Pseudomonas fluorescens strain BWKM6 was isolated on CHROMagar extended-spectrum beta-lactamase (ESBL) medium (Kanto Chemical Co., Inc., Tokyo, Japan) from the feces of M. penelope. The BWKM6 strain showed resistance to carbapenems, chloramphenicol, and tetracycline. P. fluorescens is found in a wide range of environments, including plants, soil, and water surfaces (5, 6). Different clinical strains of P. fluorescens have been reported as having high hemolytic activity, which induces cytotoxic and proinflammatory responses in epithelial intestinal cells (7, 8).

The draft genome sequence of P. fluorescens BWKM6 was analyzed by 100-bp paired-end sequencing on an Illumina HiSeq 2000 sequencing system (Hokkaido System Science Co., Ltd., Sapporo, Hokkaido, Japan). A total of 45,841,080 high-quality sequence reads was assembled *de novo* using CLC Genomics Workbench v6.5 (CLC Bio, Cambridge, MA). Approximately 99.2% of the sequenced reads were mapped again to the contigs. The final assembly of the genome produced 6,008,217 bp in 69 contigs, with an *N*_50_ value of 169,915 bp and a GC content of 60.1%. The assembled contigs were functionally annotated using the Rapid Annotations using Subsystems Technology (RAST) server (9). The genomes contained 5,335 putative coding sequences and 58 RNA genes.

The genome of BWKM6 encoded a metallo-beta-lactamase and a class C beta-lactamase. In addition, the genome encoded two multidrug-resistance proteins, streptothricin acetyltransferase and the fosfomycin resistance protein. It also encoded the following multidrug-resistance efflux pumps: the resistance-nodulation-division efflux system membrane fusion protein/inner membrane transporter/outer membrane lipoprotein (CmeA, CmeB, and CmeC), the multidrug and toxic compound extrusion family of multidrug resistance efflux pumps, the MexC-MexD-OprJ multidrug efflux system, the MexE-MexF-OprN multidrug efflux system, the macrolide-specific efflux protein MacA, and the macrolide export ATP-binding/permease protein MacB.

These results suggest that M. penelope can spread multidrug-resistant P. fluorescens through migration between Japan and eastern Siberia and that the bacteria can be transmitted from birds to humans and vice versa. The genome of P. fluorescens BWKM6 will facilitate our understanding of the ecology and global spread of multidrug-resistant P. fluorescens via migratory birds (10–12).

### Accession number(s).

The draft genome sequence of P. fluorescens strain BWKM6 has been deposited in DDBJ/EMBL/GenBank under the accession number PPHS00000000.
